# Single-target detection of *Oncomelania hupensis* based on improved YOLOv5s

**DOI:** 10.3389/fbioe.2022.861079

**Published:** 2022-08-31

**Authors:** Juanyan Fang, Jinbao Meng, Xiaosong Liu, Yan Li, Ping Qi, Changcheng Wei

**Affiliations:** ^1^ Department of IT Management, Woosong University, Daejeon, South Korea; ^2^ Department of Mathematics and Computer Science, Tongling University, Tongling, Anhui, China; ^3^ The Centers of Disease Control and Prevention, Tongling, Anhui, China

**Keywords:** the YOLOv5 algorithm, the YOLOv5, method of coordinated attention, target detection, effective channel attention mechanism

## Abstract

To address the issues of low detection accuracy and poor effect caused by small *Oncomelania hupensis* data samples and small target sizes. This article proposes the *O. hupensis* snails detection algorithm, the YOLOv5s-ECA-vfnet based on improved YOLOv5s, by using YOLOv5s as the basic target detection model and optimizing the loss function to improve target learning ability for specific regions. The experimental findings show that the snail detection method of the YOLOv5s-ECA-vfnet, the precision (P), the recall (R) and the mean Average Precision (mAP) of the algorithm are improved by 1.3%, 1.26%, and 0.87%, respectively. It shows that this algorithm has a good effect on snail detection. The algorithm is capable of accurately and rapidly identifying *O. hupensis* snails on different conditions of lighting, sizes, and densities, and further providing a new technology for precise and intelligent investigation of *O. hupensiss* snails for schistosomiasis prevention institutions.

## 1 Introduction

Schistosomiasis is a disease caused by the parasitism of pathogenic schistosome in the human blood circulation system. *Schistosoma mansoni, S. japonicum, S. haematobium, S. intercalatum*, and *S. mekongi* are the five main species of schistosomes that live in humans (Huang, 2018). Among them, *S. japonicum* is mainly distributed in China, Indonesia and the Philippines. *Oncomelania* snails is the intermediate host of *S. japonicum* in China. *Oncomelania* snails and *S. japonicum* have a very subtle interaction. There must be *Oncomelania* snails where there is an epidemic of *S. japonicum*. Usually, there is S. japonicum without Oncomelania snails, it is unlikely to generate an epidemic. *Oncomelania* snails control is a critical component of schistosomiasis eradication. By the end of 2020, China’s *Oncomelania* snails covered around 2.06 billion m^2^ (Zhang et al., 2021a). Traditional *O. hupensis* snails surveys rely heavily on the naked sight to identify based on their morphology, which is time consuming, difficult, and not very accurate (Jiang and Yang, 2020), which impairs the accuracy of the *O. hupensis* snails situation assessment. As a result, research into novel technologies and methods of *O. hupensis* snails survey should be bolstered, and the efficiency of *O. hupensis* snails survey should be increased to meet the demand for *S. japonicum* elimination efforts.

The current research on the *O. hupensis* is mainly about its breeding environment distribution, monitoring methods, control technology, etc. Hong et al. (Hong et al., 2004) and Hang and ZhouHong (2004) found that the development of *Oncomelania* eggs, oxygen consumption, enzyme activity, etc. The temperature which too high or too low is not conducive to the survival, reproduction and life span of the *Oncomelania* snails. The most suitable temperature for the survival of the snails is 20–30°C (Huang, 2006). He et al. (2006) analyzed the number and spatial distribution characteristics of *Oncomelania* in different land types in a typical study area through indoor tests and field surveys. The results showed that there were large differences in the density of live *O. hupensis* between different land types. Yang et al. (2003) introduced the methods of microbial *O. hupensis* control, describing the general microbial *O. hupensis* control tests and their effects. The results of the microbial tests of *Pseudomonas conrexa chester*, *Streptomyces griseolus* 230, *Streptomyces diastatochromogeryes* 218, etc., have a strong role in killing *O. hupensis* and *O. hupensis* eggs. Wang (2010) extracted geometry, edge morphology, and brightness features from *O. hupensis*. They established classifiers with neural network techniques to increase recognition accuracy and stability. Shi et al. (2021a) developed a visual intelligent recognition model for *O. hupensis* using deep learning technology. They proposed a convolutional neural network with an optimized training strategy of “Data Augmentation + Transfer Learning,” which is capable of accurately recognizing *O. hupensis* images.

In summary, the algorithm used in the existing research is a two-stage target detection algorithm, which first generates a target pre-selection frame, and then classifies and regresses the area through the CNN network layer to obtain the detection frame (Xie et al., 2022a). The YOLO algorithm used in this study can generate the predicted classification probability and predicted coordinate value of the detected object with only one detection, so that it has faster training speed and detection speed than previous research algorithms. The *O. hupensis* snail is a small target with fuzzy border characteristics; its appearance is complex and difficult to recognize, and it presents unique detection issues. The results indicated that YOLOv5s-ECA-vfnet improved the algorithm’s precision (P), recall (R), and mean average precision (mAP) by 1.3 percent, 1.26 percent, and 0.87 percent respectively, over YOLOv5s, thereby providing a new technology for accurate and intelligent investigation of *O. hupensis* snails.

## 2 Algorithm YOLOv5

Three components make up the YOLOv5 algorithm. The first section contains the input, which consists of a 608-part training image. The second section is the backbone network, which extracts information-rich features from the input photos using the CSPDarknet53 network. The third section is the detection layer, which employs multiple scales for detection (Cai et al., 2021a). In addition, it incorporates a new bottom-up path aggregation network structure (Path Aggregation Networks, PAN) following the Feature Pyramid Networks (FPN) structure (Lin, 2016) to achieve feature information fusion at various scales. Following that, predictions are made on the three created feature maps.

**FIGURE 1:**
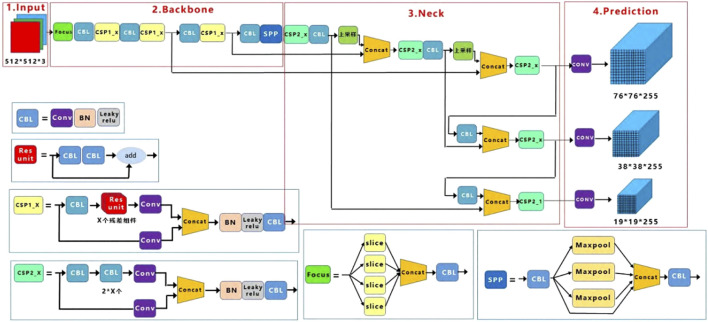
The overall algorithm structure of YOLOv5.

The YOLOv5 algorithm’s convolution kernel is primarily 33 or 11, and the convolution structure is composed of a convolution layer, a batch normalization (BN) layer, and an activation function layer (Wu et al., 2018; Zhang et al., 2021b; Zhou et al., 2021). For multi-scale fusion, the Spatial Pyramid Pooling (SPP) structure employs maximum pooling of 1 × 1, 5 × 5, 9 × 9, and 13 × 13 (Cui et al., 2019; Gao, 2020). Furthermore, YOLOv5 maintains a multi-scale detection framework. After the backbone network extracts the features, two upsampling and three convolutions are done to achieve significant and tiny target categories and position prediction at three scales of 19 × 19, 38 × 38, and 76 × 76, respectively. The YOLOv5 makes advantage of adaptive anchor frame computation, which determines the optimal anchor frame values in the training set based on the training data set (Shu and Zhang, 2021). Figure 1 illustrates the overall algorithm structure of YOLOv5.

The YOLOv5s is a fast and precise detection method that performs well on open-source datasets, but its detection performance for *O. hupensis* identification tasks still needs to be improved. Experiments and tests have confirmed the enhanced algorithm described in this paper’s effectiveness.

## 3 Improvement of YOLOv5s-ECA-vfnet *O. hupensis* detection algorithm

### 3.1 YOLOv5s-ECA network based on effective channel attention mechanism

**FIGURE 2:**
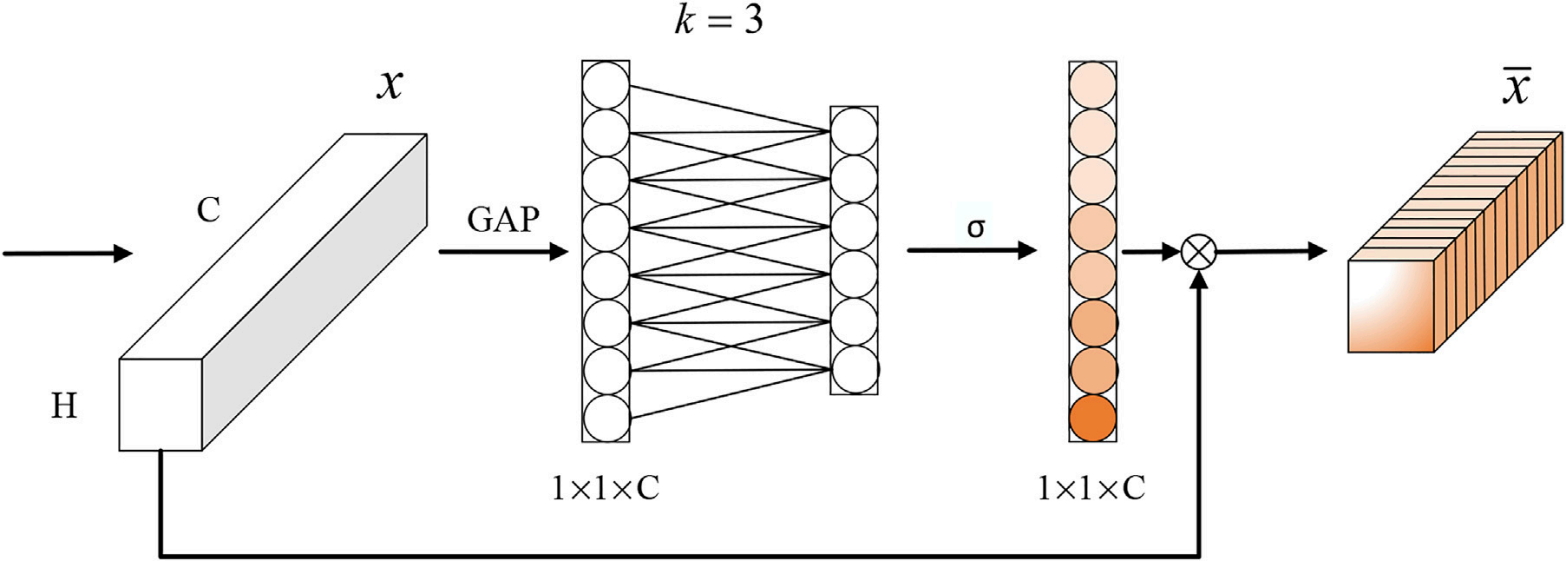
ECA attention module structure diagram.

When the YOLOv5s network extracts features, it treats all model channels equally, which limits the algorithm’s detection effectiveness somewhat. Due to the study’s small and densely dispersed *O. hupensis*, an effective channel attention mechanism is incorporated into the CSPDarknet53 feature extraction network of YOLOv5s. The enhanced model is dubbed YOLOv5s-ECA-vfnet. The attention mechanism has been demonstrated to be a critical component of enhancing target detection performance and is frequently used in a variety of popular detection algorithms (Cui et al., 2021a; Hu and Yan, 2021). The most prominent feature of Efficient Channel Attention is that it avoids downscaling and cross-channel interactions, while reducing the complexity of the model and enhancing feature representation (Cai et al., 2020; Wang et al., 2020; Zhu et al., 2021). Figure 2 illustrates the structure of the ECA’s attention module.

The ECA module generates channel attention *via* a fast 1D convolution of size k, the size of which is completely determined by the adaptive channel dimensionality correlation function. After importing feature images with constant dimensionality χ , all channels are globally averaged and pooled. The ECA module will learn features using a one-dimensional convolution that can share weights, and will involve k nearest neighbors per channel to capture cross-channel interactions when learning features. The adaptive k value is determined by the proportional relationship between the masked area of cross-channel information interactions and the channel dimension C, as shown in Eq. 1.

$$\text{K}=\left(\text{φ}\right)={\left|\frac{\text{log}2\left(\text{C}\right)}{\text{γ}}+\frac{\text{b}}{\text{γ}}\right|}_{\text{ood}}$$ (1)

where γ and b are set to 2 and 1 by default, and ${\left|\text{*}\right|}_{\text{ood}}$ denotes the nearest odd number, and C is the channel dimension.

The general channel attention mechanism module selects a high-level feature map for global pooling and then compresses the two-dimensional features of each channel using dimensionality reduction. After this computation, the entire feature can be considered compressed in order to obtain a complete global perceptual field, but some spatial feature information is lost as the dimensionality is reduced. The relationship between the channel and spatial dimensions cannot be reflected. While more mature CBAM attention mechanisms are based on both space and channel, their computational processes are completely independent, significantly increasing computational time and effort (Wang and Wang, 2021). In comparison, the important significance of Efficient Channel Attention is: Firstly, it is not required dimensionality reduction. all channel and spatial dimension information will be integrated without losing information; secondly, the coverage area and channel dimension through the interaction of cross-channel information. The proportional relationship of C obtains an adaptive k value, which reduces the amount of calculation. The model’s complexity is reduced while the expression of features is increased, increasing the model’s accuracy (Xie et al., 2022b).

### 3.2 Optimization of the anchor frame parameters

The YOLOv5 implements the concept of Auto Learning Bounding Box Anchors, which utilizes the K-Means algorithm to automatically calculate the appropriate anchor box based on the labeled target box (ground truth) (Li et al., 2021a; Cui et al., 2021b), and the anchor box is learned from the training data.

Since the size of the detection target of the custom dataset differs from that of the public dataset, YOLOv5 learns the size of the anchor frame by relearning it automatically. The YOLOv5 generates nine anchor boxes from the COCO dataset: (10, 13), (16, 30), (33, 23), (30, 61), (62, 45), (59, 119), (116, 90), (156, 198) and (373, 326). In this paper, we add three anchoring frame sizes for detecting small targets with inconspicuous boundaries, namely (5, 6), (8, 14), and (15, 11). Anchor boxes were assigned based on the detection layer scale, and the statistical anchor box assignments for detecting *O. hupensis* with small targets are shown in Table 1.

**TABLE 1:** Anchor Box allocation table.

Feature map	20*20	40*40	80*80	160*160
Receptive field	Large	Larger	Medium	Small
Anchor boxes	(116,90)	(30,61)	(10,13)	(5,6)
	(156,198)	(62,45)	(16,30)	(8,14)
	(373,326)	(59,119)	(33,23)	(15,11)

### 3.3 Improvement of the confidence loss function

The YOLOv5 network uses the focal loss function, which has similar loss weights for positive and negative samples superimposed, and the focus is on more difficult to detect targets, which creates some difficulties when training the pinned *O. hupensis* snails. The number of positive and negative samples is highly unbalanced due to the small size of the pinned *O. hupensis* snails target. If both positive and negative sample losses are treated equally, the network’s convergence speed will be slowed and the learning information about the target will be lost (Li et al., 2021b). The original loss function concentrates the network’s attention on low-quality samples, reducing the efficiency of updating the target detection model parameters and having a direct effect on subsequent detection results. When the target arrangement is dense, the non-maximum suppression algorithm will eliminate the interference of redundant prediction boxes according to the highest scoring prediction box. At this time, the high score prediction box will affect the subsequent screening results. In this paper, we introduce the VariFocal Loss (Ye, 2021) function for YOLOv5s, as defined in Eq. 2

$$\text{VFLoss}\left(\text{p},\text{q}\right)=\{\begin{array}{cc} -q\left(q\log\left(p\right)+\left(1-q\right)\log\left(1-p\right)\right) & q>0\\ -\alpha {\text{p}}^{\text{γ}}\log\left(1-p\right) & q=0 \end{array}$$ (2)

In which p is the confidence level of the network output prediction frame, and  q is the target IoU score. For positive samples with higher scores in training, the model increases their share of the loss weights. For negative samples in training, q is 0. The negative sample loss is incorporated with a γ downgraded weight, while the positive sample loss is not introduced in the calculation of γ. The weight of the negative sample loss can be effectively reduced without affecting the positive sample weight. In order to balance q, α downscaling is used and ultimately allows the network to focus its training on the positive samples with high scores.

## 4 Finds of experiments and their analysis

### 4.1 Platform for experimentation

The deep computing platform used in this paper is comprised of an Intel^®^ Core(TM) i7-8700K CPU at 3.20 GHz as the central processor, Windows 10 Pro as the operating system, 16 GB DDR4 as the running memory, a GeForce GTX 1070 8 GB as the graphics processor, Python 3.8 with Pytorch 1.9 as the programming language.

### 4.2 Experimental dataset

**FIGURE 3:**
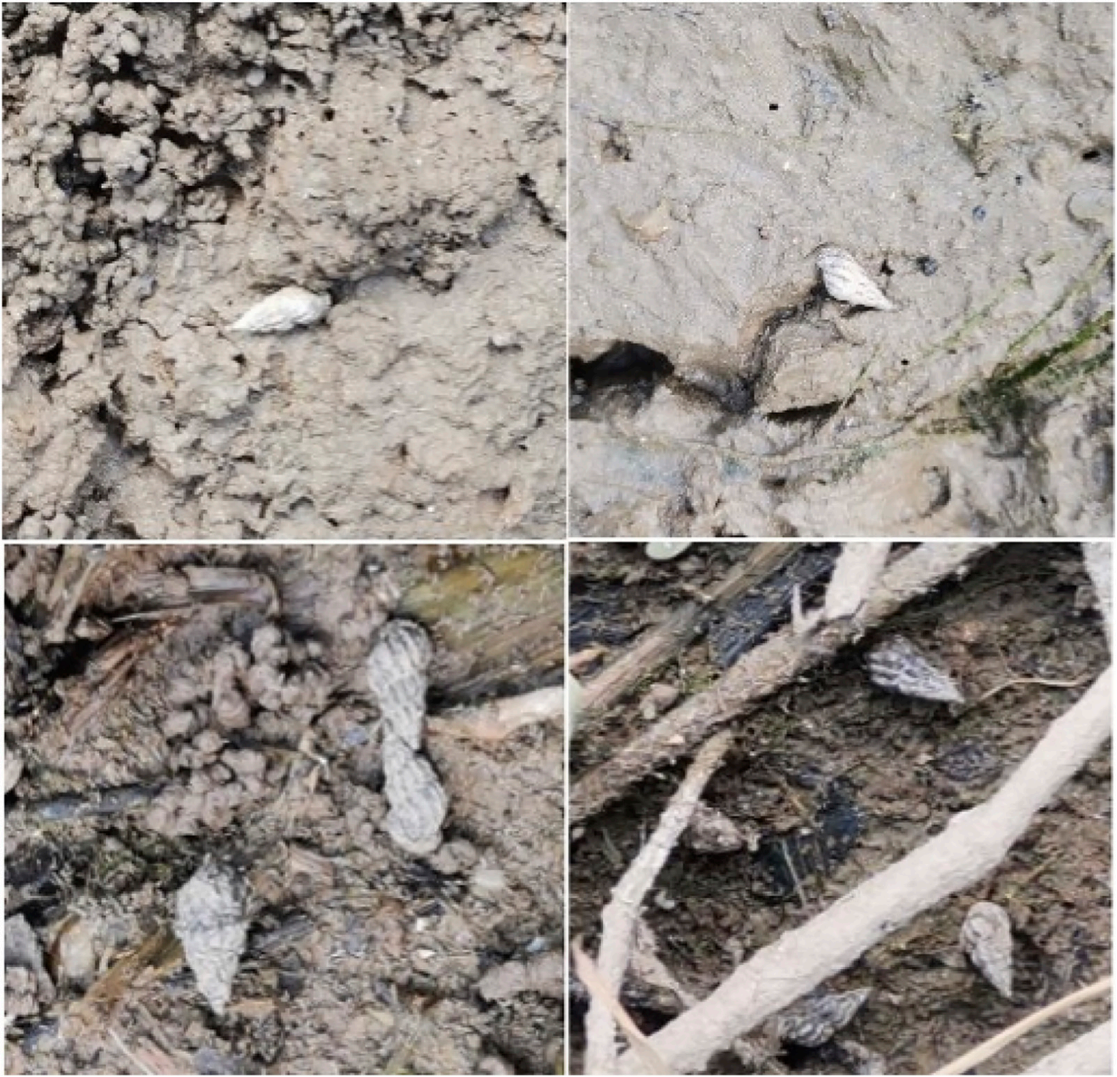
Data set sample.

The data in this paper were obtained from the study site: Jianghuzhou Island in Zongyang County on the Yangtze River Island in Zongyang County, the north bank of the lower Yangtze River in Anhui Province, China, a dataset of 2000 images of lake and the *O. hupensis* snails were built under the guidance of blood control professionals from the Tongling Center for Disease Control and Prevention according to the distribution pattern of *O. hupensis* snails (Zhou, 2019). Some samples of the dataset are shown in Figure 3.

**FIGURE 4:**
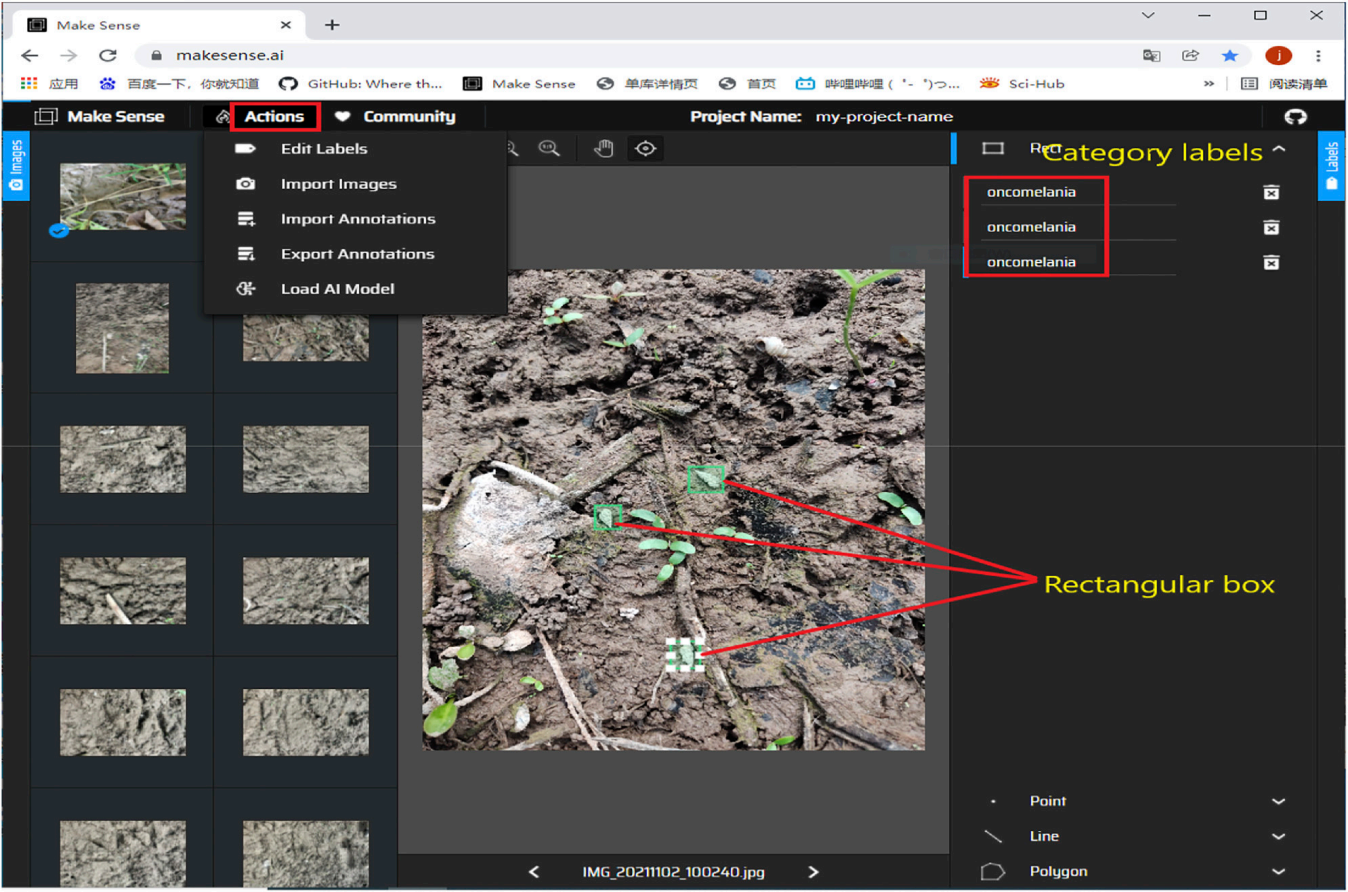
Example of data set annotation.

The collected data were filtered and organized, and all *O. hupensis* image samples in the collected images were annotated using the marker software Make-Sense. The annotated images’ annotation information was saved in the form of.txt files, which included the target object’s category and coordinate information. Figure 4 illustrates a data annotation example.

### 4.3 Evaluation indicators

The average precision (AP) and mean value of average precision mAP are frequently used in target detection to assess the model’s detection effectiveness and performance (Zhao et al., 2021). AP is defined as the area under the Recall and Precision curves, which is equivalent to mAP in this study for a single target. Ratio of area intersections (IoU). The model’s ability to predict the location is determined by comparing the area of the rectangular area predicted by the model to the rectangular area calibrated in the validation set. Precision (P): The ratio of correct targets detected by the model to the total number of targets, which indicates the model’s detection accuracy (Cai et al., 2021b; Shanid and Anitha, 2021).

$$\text{P}=\frac{\text{TP}}{\text{TP}+\text{FP}}$$ (3)

Recall R: Recall is the ratio of targets detected by the model to the total number of targets, and it indicates the model’s ability to identify a wide variety of targets.

$$\text{R}=\frac{\text{TP}}{\text{TP}+\text{FN}}$$ (4)

$$\text{AP}=\int_{0}^{1}\text{P}\left(\text{R}\right)\text{dR}$$ (5)

Where TP (True positive) denotes the number of positive samples correctly detected, that is, the prediction frame belongs to the same category as the labeled frame and IoU >0.5. FP (False positive) denotes the number of positive samples incorrectly detected. FN denotes the number of negative samples incorrectly detected. Since precision and recall are affected by confidence level, evaluating model performance solely on the basis of precision and recall would be unscientific and limited. Therefore, in our experiments, we use Average Precision (AP) to evaluate the model’s recognition performance, which is one of the most important indices for evaluating the performance of mainstream target detection algorithms.

### 4.4 Training parameter design

The spiked data set is divided randomly into training sets and test sets in a 7:3 ratio. There are 1,400 training and 600 test sets, respectively. The scale of the input image is 640 × 640. The number of training batches is 8. The training momentum is 0.843. The initial learning rate is set to 0.0032. The weight decay is set to 0.00036. The training epochs for theYOLOv5s and the YOLOv5s-ECA-vfnet are both 400 according to the model’s characteristics.

### 4.5 Analysis of findings

#### 4.5.1 Training process loss values

The loss function value in deep learning can reflect the error between the final prediction result of this target detection model and the actual true value, and is used to analyze and judge the training process’s merit, the model’s convergence, and whether it is over fitted (Wang and Fu, 2018). While the loss function can be considered a layer of the network within the model definition in the PyTorch framework used in this paper. In actual use, it is more focused on the forward propagation process as a functional function (Gong et al., 2019; Fang et al., 2022; Zhang et al., 2022).

**FIGURE 5:**
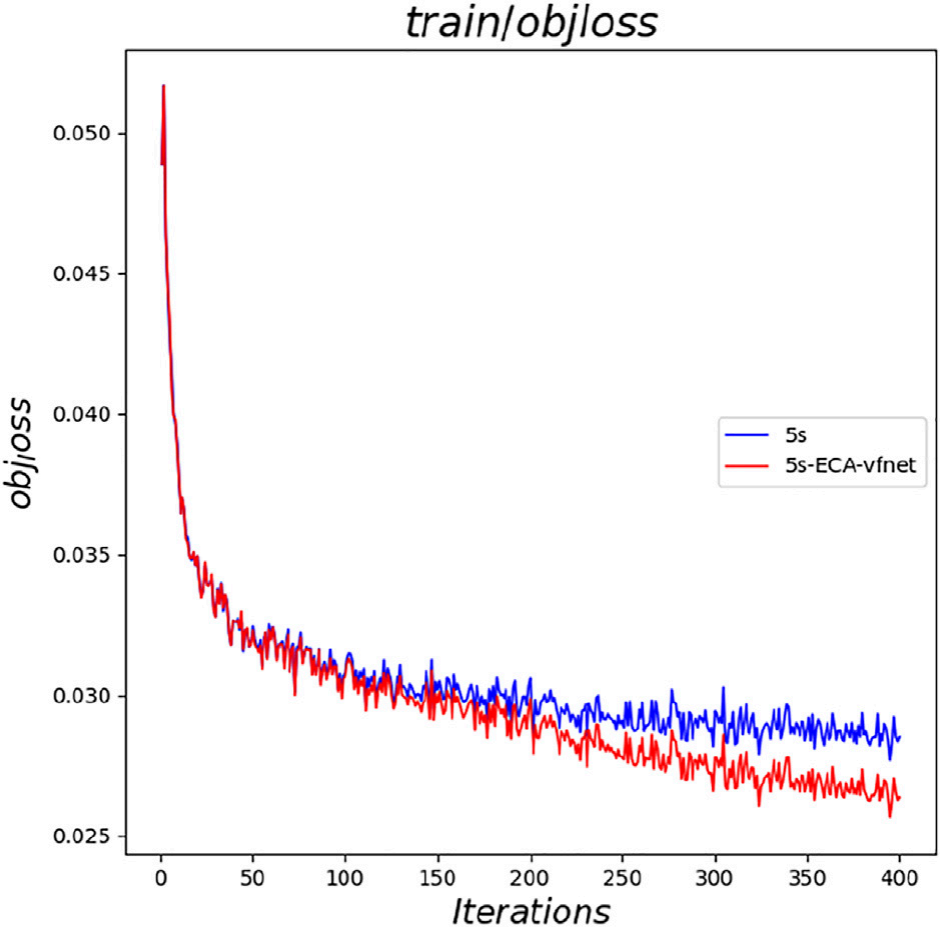
Curve of the loss function value with the number of training rounds.

The loss function values of the original YOLOv5s model and the improved YOLOv5s-ECA-vfnet model are compared and analyzed, and the loss function values of YOLOv5s-ECA-vfnet are found to be significantly lower than those of the original YOLOv5s model after 150 epochs. Loss function as one of the criteria for evaluating the quality of model training. We found that the YOLOv5s-ECA-vfnet is significantly better than the YOLOv5s, and the YOLOv5s-ECA-vfnet has a lower loss function value under the same number of training epochs. Figure 5 depicts the loss function value’s transformation curve as a function of the number of training rounds.

#### 4.5.2 Parameter convergence results

The training and evaluation parameters typically reflect the process of developing the model and the effect of target detection (Fang et al., 2021a; Shi et al., 2021b). Box Loss, Objectness Loss, Precision, Recall, mAP@0.5, mAP@0.5:0.95, and so on are used as the primary parameters to determine the degree of convergence in this paper. The closer Box Loss and Objectness Loss values are to 0, the better the training effect. The Precision, Recall, mAP@0.5, and mAP@0.5: 0.95 values are to 1, the more converged the parameters are, and thus the better the training effect (Wang et al., 2017; Fang et al., 2021b).

**FIGURE 6:**
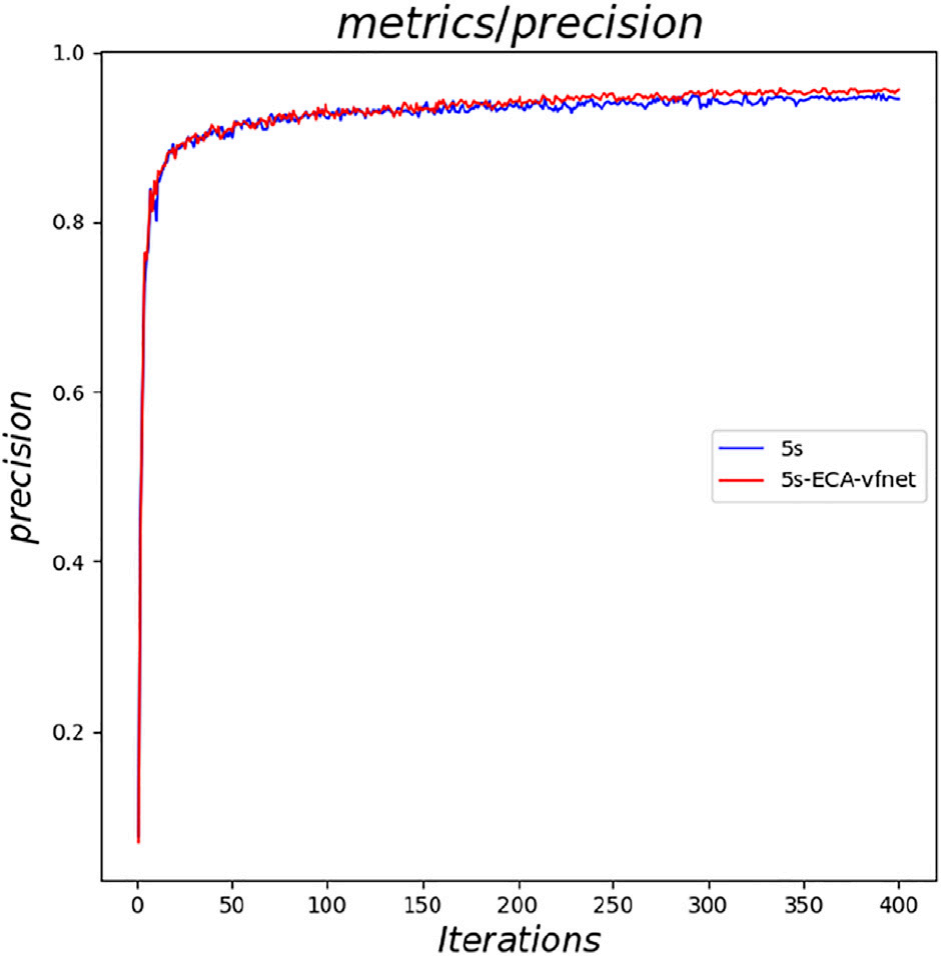
Comparison of precision.

**FIGURE 7:**
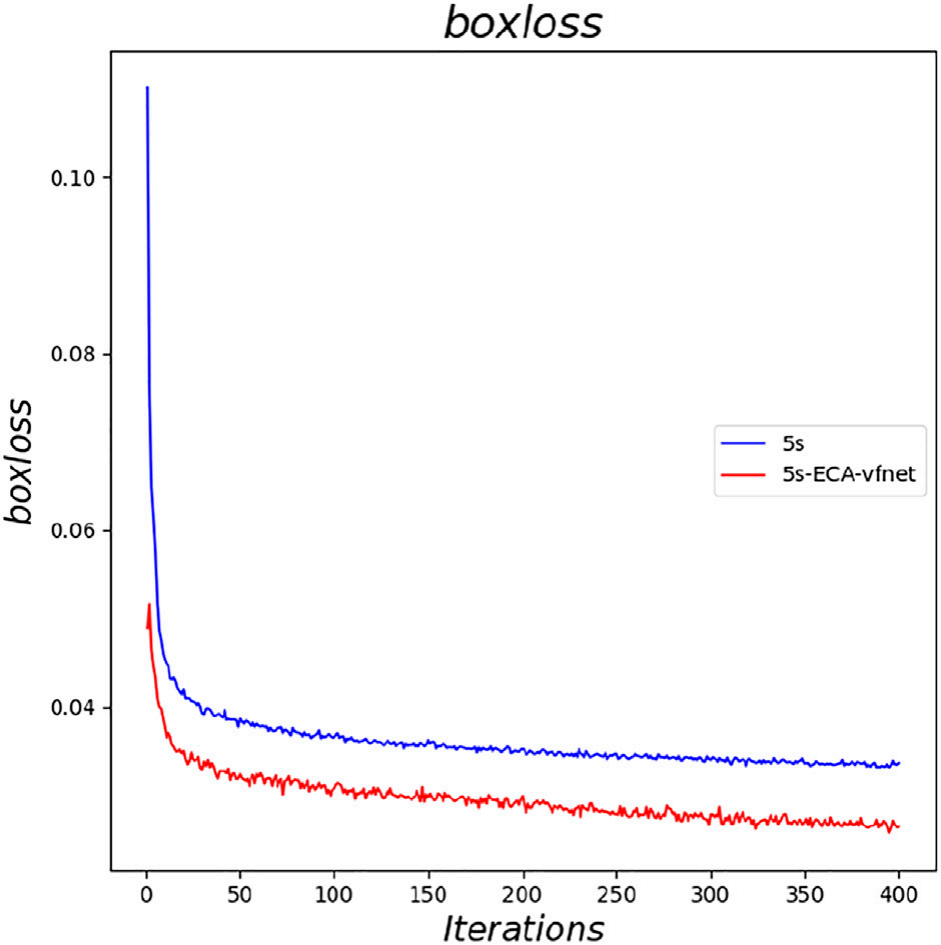
Comparison of boxloss.

**FIGURE 8:**
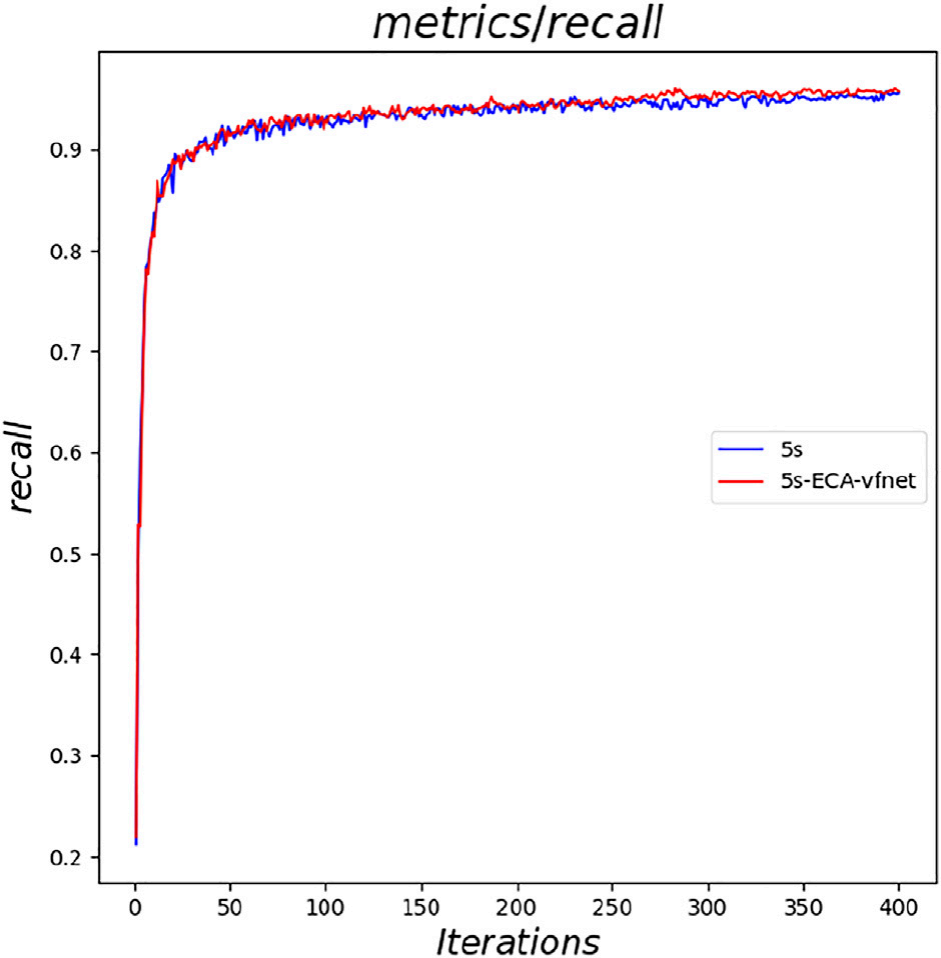
Comparison of recall.

**FIGURE 9:**
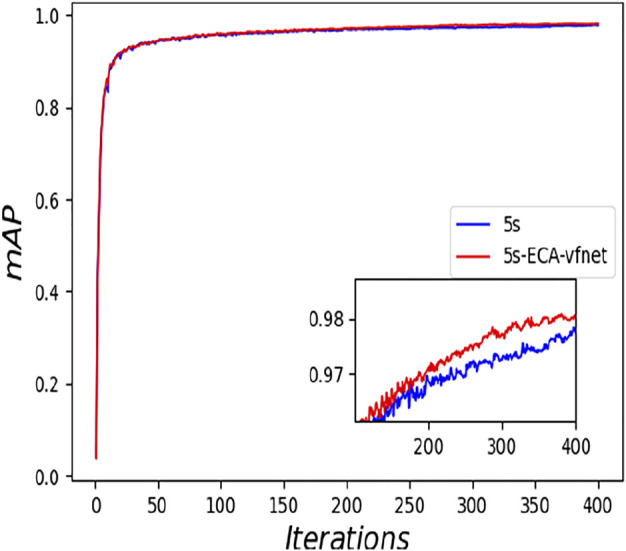
Comparison of mAP.

The experimental results indicate that the parameters of both YOLOv5s and its improved YOLOv5s- ECA-vfnet gradually converge (the loss parameter converges to 0 and the result parameter converges to 1). Figure 6 illustrates the accuracy of the YOLOv5s training process, and the improved algorithm outperforms the traditional YOLOv5s. From the loss curve of border regression in Figure 7, it can be seen that the loss value of the YOLOv5s- ECA-vfnet is significantly smaller than that of the YOLOv5s algorithm and is stable at about 0.01. Figures 8, 9 show the recall rate and the average precision mean of training, respectively, and the proposed algorithm has also improved.

In order to verify the effectiveness of the YOLOv5s-ECA-vfnet algorithm in snail detection, compared with the YOLOv5s, the experiment uses precision, mAP, Recall and inference time as evaluation indicators. The results are shown in Table 2.

**TABLE 2:** Comparison with the YOLOv5s.

Model	Images	P/%	R/%	mAP@0.5/%	Frame/Sec
Yolov5s	2000	92.48	92.89	95.51	61
YOLOv5s-ECA-vfnet	2000	93.78	94.15	96.38	55

#### 4.5.3 Predictability of the YOLOv5s-ECA-vfnet algorithm

**FIGURE 10:**
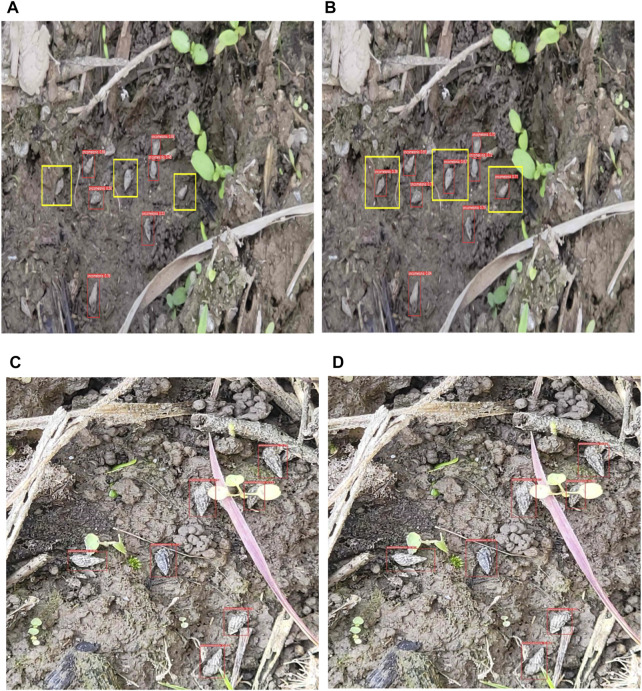
The result of object detection. **(A,C)** The prediction of the YOLOv5s; **(B,D)** The prediction of the YOLOv5-ECA-vfnet; The red rectangular boxes are the probability values of the algorithms to identify the pictures containing the snails, and the three yellow boxes are snails that YOLOv5s cannot detect in **(A)**. The improved algorithm can detect the snails in **(B)**. **(C,D)** are different in the predicted probability values.

The effect of *O. hupensis* target visualization detection is shown in Figure 10, from Figures 10A,B it can be seen that the detection of densely arranged target regions with the original algorithm is seriously missed, while the detection rate of the improved algorithm is significantly improved and the effect of dense targets on detection is effectively reduced. Figure 10C The loss function in the original algorithm does not pay enough attention to the high-quality prediction frames, resulting in a low confidence of the output prediction frames. YOLOv5-eca-vfnet is added with an attention mechanism, improved anchor frames, and improved loss function, and from Figure 10D it can be found that the confidence of the prediction frames is significantly improved, the prediction frames can optimally cover the target area, and the final output is more accurate target location information.

## 5 Conclusion

In recent years, machine learning techniques of computer vision have been gradually applied to the field of parasitic disease control research, and certain results have been achieved (Eshel et al., 2017). The recognition results are often constrained by the merits of manually selected *O. hupensis* features through the problems of difficult manual extraction of *O. hupensis* image features, poor adaptability and weak anti-interference ability. In contrast, visual recognition based on deep learning techniques can often achieve better results.

To solve the problems of complex appearance of *O. hupensis*, inconspicuous boundary features, small detection target and low accuracy of *O. hupensis* detection due to small *O. hupensis* data samples, this paper introduces the effective channel attention mechanism, propose the YOLOv5s-ECA-vfnet algorithm based on *O. hupensis* conch image detection by optimizing the anchor frame parameters and changing the confidence loss function. The experimental findings show that the YOLOv5s-CA-vfnet algorithm has improved 1.3%, 1.26% and 0.87% in Precision (P), Recall (R) and mean Average Precision (mAP), respectively, over YOLOv5s. The *O. hupensis* image classification dataset also provides a data base for the subsequent study of other parasitic host snail identification.

There are some limitations in this study. 1) The sample collection area of this study was limited to Anhui Province, and could not represent the image recognition ability of samples from other regions of the country. 2) This study established an *O. hupensis* snail identification model, which could only identify *O. hupensis* snail, but could not sub-classify and identify which subspecies of *O. hupensis* snail. In the future, we will further expand the scope of *O. hupensis* snail and similar snail sample collection and further optimize the subclassification capability of the intelligent model. Further increase the scope of application of the model (Rauf et al., 2020; Sharifizadeh et al., 2021).

## Data Availability

The original contributions presented in the study are included in the article/Supplementary Material, further inquiries can be directed to the corresponding authors.
